# Paralysis Agitans

**Published:** 1886-06

**Authors:** Edward C. Mann

**Affiliations:** Superintendent Sunnyside Home for Nervous Invalids; 34 West 33d street, N. Y.


PARALYSIS AGITANS. *By* EDWARD C. MANN, M.D., *Superintendent Sunnyside Home for Nervous Invalids.*

Paralysis agitans is a disease characterized by involuntary tremors or shaking movements of the limbs, head or trunk of the body, of a spasmodic nature, by diminished muscular power or paresis, and in an advanced stage of the disease by a disturbance of equilibrium, shown by a tendency to stoop forward, and an accelerated walk. The tremors constitute the most distinctive features of the disease. In the first stage of the disease the tremors and paresis affect either the arm and hand or perhaps the leg or the head. Secondarily, the tremors become generalized and finally affect the whole body. The disease is very insidious and progressive and the tremors tend, as the disease advances, toward convulsive activity. Sleep relieves the patient, as then there is a cessation of the convulsive tremors. The forward stoop is caused by paresis of the extensor muscles of the back and the muscles become rigid and contracted as do also the muscles of the extremities. There is a verbal paralysis to some extent. If the disease progresses to a fatal termination the patient wakes up greatly frightened in the night by the convulsive agitation appearing even then, and the poor sufferer is finally completely exhausted. The cutaneous sensibility is not affected in paralysis agitans, neither as a rule are the special senses, nor the integrity of the intellect. The disease comes on in advanced life generally. All writers upon diseases of the nervous system concur in the statement that, with a few rare exceptions, it is incurable.

Dr. J. Russell Reynolds, of England, has reported one very interesting case of cure by the continuous or galvanic current, and Remak has also reported one. The diagnosis is easy, as in other tremors the trembling does not occur when the parts are not in use and are supported. The non-senile form may occur in early life, but there is no fatal tendency and the tremors disappear, except when voluntary movements are attempted, and there is no disturbed equilibrium or forward stoop. Hysterical paralysis agitans is accompanied by other hysterical symptoms. Intermittent paralysis agitaus in the young is removed by anthelmintics, as it generally depends on intestinal worms. Chorea is a widely different disease, and is not liable to be confounded with paralysis agitans. The lesion in the fatal cases would seem to consist progressively of irritation, congestion and, finally, sclerosis and atrophy of the motor nerve centers, while in the cases on record that have recovered the trouble was probably functional, and had not progressed beyond the stage of impaired nutrition, irritation and congestion of the motor nerve centers. It is stated that an atrophic condition of the spinal cord, pons varolii, crura or medulla oblongata has been found in several careful dissections of those in whom the disease has proved fatal, and also sclerosis and patches of gray degeneration. It is possible that in some cases *multiple sclerosis* may be mistaken for paralysis agitans. The former disease, however, rarely occurs after thirty years of age and generally between twenty and twenty-five years, and is more common in women than in men, while the latter disease generally attacks the male sex more frequently. The earliest symptoms of multiple sclerosis is rhythmical tremors, but this trembling generally disappears as paralysis supervenes. The eyes are involved, the vision being indistinct and double vision occurring. The speech is slow and drawling; vertigo is a marked symptom; paresis of the lower limbs, terminating in true motor paralysis, is a marked symptom. There is contraction of the limbs; the general expression changes and the mind becomes weakened, and finally death occurs from failure of the nutritive function. It will be seen that, aside from the tremblings of head, neck, trunk and limbs, which come on *only when the muscles are being exerted*, in disseminated sclerosis, the two diseases have no feature in common. The following case, interesting by reason of the rarity of recoveries, occurred in the person of a Mrs. E., aged 50, resident of New York city and the superintendent of one of our charitable institutions, and it serves to illustrate these cases very well. The disorder had come on gradually, as the result of domestic unhappiness and grief, and had finally culminated in a condition of sub-acute mania, complicating the case very seriously. At first I declined to take such a case, and gave the ladies who were interested in the patient a very unfavorable prognosis concerning it. I finally agreed to treat her for one month, her friends agreeing to remove the patient at the expiration of that time if I could effect no improvement, which I certainly did not expect to be able to do. At the time of her admission the trembling was incessant and involved all the limbs. There were delusions of suspicion and dread and fear of persecution—in other words, marked mental disorder. There were hallucinations of sight and hearing. There would be exacerbations of the tremblings, due to emotional disturbance. There was marked muscular rigidity and contraction, so that the head was thrown forward and fixed, and the trunk was also bent forward.

Walking seemed very difficult and also talking. The muscular force and the cutaneous sensibility were normal, so far as I could ascertain. There was marked tremulousness of the tongue when protruded. The trembling at first attacked one hand and arm, and gradually spread all over the body. I regarded my patient as hopelessly incurable. I however directed warm baths with cold affusions to the head at night, opened the bowels freely with a mercurial cathartic followed by salines, and then put my patient practically on a milk diet and secluded her from all society save that of her nurse, and directed the latter to administer three times a day drachm doses of sodium bromide and tincture of hyoscyamine, and I soon found to my surprise that my patient was improving very much. Electricity in the form of central galvanization and also a bi-temporal current were employed. The mental excitement soon began to disappear, the muscular trembling gradually subsided very much in proportion as the mind became quiet, and at the end of one month I saw that my patient was rapidly improving, contrary to all my expectations. I accordingly allowed her to take moderate exercise in the open air and put her on a full diet. The rigidity and contraction of the muscles disappeared gradually, the gait becoming assured, the head coming up erect and also the trunk. The speech lost its tremulousness and the face assumed a much more bright and intelligent expression. At the end of the second month all mental disturbance had passed away, the mental faculties remaining normal. I now discontinued the use of the sodium and hyoscyamine and also the central galvanization, substituting instead the induced current in the form of general Faradization, using it as a nervous stimulant and tonic. I considered that by the constant current I had removed the nutritive defect in the central nervous system, improving the tone and nutrition, not alone of the brain and cord, but also of all the deeper tissues of the body. A tonic mixture containing quinine, phosphorous and strychina was now ordered and the patient's weight increased markedly during the third month of her treatment. At the expiration of the third month she was discharged perfectly well, not a trace of trembling being visible in muscular action, speech or gait. The mental faculties were pefectly restored. The patient, against my advice, returned to her laborious post of duty and has since remained perfectly well. I do not know that I should in another case get such a favorable result. I am afraid not, but should certainly work with more expectation of such result than I have previously entertained. My success in this case, however, warrants me in expressing the hope that such cases may have the benefit of long-continued applications of electricity, with bromide of sodium in drachm doses in half a tumbler of water thrice daily, between meals, and either the fluid extract of hyoscyamine, the tincture or hypodermic injections of $\frac{1}{50}$ gr. of the crystallized extract of hyoscyamine. Prof. Charcot considers it probable that the morbid anatomy of many of the cases that go on to a fatal termination, consists of obliteration of the central canal of the cord by increase of its epithelial lining, overgrowth of the nuclei which surround the ependyma and marked pigmentation of the nerve cells, principally those of Clarke's posterior vesicular columns. In my case, if the paralysis agitans had depended upon an atrophic condition of the spinal cord, pons varolii, crura or medulla oblongata, or, in other words, had depended on organic changes, I do not think a cure could have been obtained. On the other hand, I am inclined to think that if there was degeneration, due to the new formation of connective tissue compressing the cord and nerve structures, the constant current, *perhaps*, by its catalytic effect could have had the power to remove such new formation, freeing the compressed nerve structure. My case, moreover, may have depended on congestion of the nervous substance or the membranes of the upper part of the medulla spinalis, oblongata and pons, which had not gone on to sclerotic atrophy and the galvanic current unquestionably would have relieved that condition permanently. I not only had to combat disease of the motor centers but also of the intellectual centers. The disease, I presume, commenced in the motor tract of the cerebrum, since the arm was first affected, and soon the corresponding one. I considered my case, however, probably to have been one in which there was congestion, weakness and irritability and instability of the molecular nerve structure of the nerve centers, owing, perhaps, to mal-nutrition, and that the disease was functional in character rather than organic. If so, then we may say that there are curable functional cases of paralysis agitans. My patient had not a rheumatic diathesis or any other morbid diathesis which could have disposed her to the disease. The case was to me exceedingly interesting.

Dr. J. Russell Reynolds reported in the London *Lancet* of Dec. 3d, 1859, a case of paralysis agitans cured by the constant or galvanic current. The patient was a married man and father of twelve children, aged 57, carpenter by trade. For the five years previous to Dr. Reynolds seeing him, he had suffered much anxiety respecting his children, although

Dr. Reynolds did not feel certain that that was the cause of the paralysis agitans. Occasional tremor of the right arm and leg appeared first about two years previous to treatment, and seemed to be increased by anything requiring expenditure of nervous power. These symptoms had much increased just before treatment was commenced by Dr. Reynolds. The arm was worse than the leg, and in it the motion most constant and most extensive was that at the shoulder joint. The involuntary movement of the arm could be arrested by the patient lying on the sofa and pressing the forearm against the ilium; but any attempt to move the limb voluntarily at once reproduced the shaking, although the patient remained in the recumbent position. The movement was instantly arrested when the doctor firmly grasped either the forearm in any part of its upper two-thirds, or the arm in its lower third. This did not seem to be a mere mechanical arrest of the movement, for it could not be effected by holding the wrist; and the jerking recommenced if, while the extremity was grasped in the manner described, the patient made any attempt at a voluntary movement. The mental condition of the patient and his general health appeared unaffected. The sensibility was unchanged in the right upper extremity, there was no deviation of the tongue nor distortion of the features. The patient could walk well, and without dragging either leg. A continuous galvanic current was applied to the arm and forearm, the movements of the latter being at the time arrested by pressure. At the end of five minutes he could execute voluntary movements without the least tremor, and emotional excitement failed to reproduce the jerking. The temperature of the two arms, examined after the current had been passing for half an hour, was equal. The involuntary movements did not return until three hours after the current was discontinued, they then reappeared and continued throughout the evening; stopped at night, but returned on the following morning.

"Oct. 6. The current was applied while the arm was in violent movement, but in two minutes it became perfectly still: application continued for one hour.

"Oct. 7. Last evening there was no jerking nor tremor for five hours after the current was discontinued; then it commenced, but stopped spontaneously in about half an hour, and during the remainder of the evening there was nothing more than very trifling tremor. The jerking has returned this morning, but it is much less than on the first day of observation. There were but 20 alternations in fifteen seconds, and the range of movement is from three to four inches. The movement, therefore, is only 86 feet per second—less than one-eighth of what it was three days ago. The current was applied on the 7th, on the 8th and 10th, and after the 10th—*i. e.*, after five applications—the spontaneous jactitation completely ceased. When any weight is held in the hand and it is lifted towards the mouth, there is tremor, but this is slight, is not more than has occurred for the last two years, and it immediately ceases when the effort is discontinued. The arm and hand are weak; every movement can be executed by them voluntarily, but such movements are feeble.

"28th. Has written me a letter in a good and legible hand. The current was applied about every other day for an hour until Nov. 10th, and during this time there was steady increase in the power of the limb and the jactitation did not return. No medicine of any kind was given.

"Nov. 12th, quinine and gin was ordered.

"15th. W. F. is in perfect health; there is no jactitation and only the slight tremor already described, when the hand with something in it is raised toward the mouth. The current employed in this case was from Pulvermacher's chain battery of 120 links."

34 West 33d street, N. Y.

